# Comparison of ablation defect on MR imaging with computer simulation estimated treatment zone following irreversible electroporation of patient prostate

**DOI:** 10.1186/s40064-016-1879-0

**Published:** 2016-02-29

**Authors:** Govindarajan Srimathveeravalli, Francois Cornelis, Joseph Mashni, Haruyuki Takaki, Jeremy C. Durack, Stephen B. Solomon, Jonathan A. Coleman

**Affiliations:** Interventional Radiology Service, Department of Radiology, Memorial Sloan Kettering Cancer Center, 1275 York Avenue, New York, NY 10065 USA; Department of Radiology, Pellegrin Hospital, Place Amélie Raba Léon, 33076 Bordeaux, France; Urology Service, Department of Surgery, Memorial Sloan Kettering Cancer Center, 1275 York Avenue, New York, NY 10065 USA

**Keywords:** Computer simulation, Prostate, Ablation, Irreversible electroporation

## Abstract

To determine whether patient specific numerical simulations of irreversible electroporation (IRE) of the prostate correlates with the treatment effect seen on follow-up MR imaging. Computer models were created using intra-operative US images, post-treatment follow-up MR images and clinical data from six patients receiving IRE. Isoelectric contours drawn using simulation results were compared with MR imaging to estimate the energy threshold separating treated and untreated tissue. Simulation estimates of injury to the neurovascular bundle and rectum were compared with clinical follow-up and patient reported outcomes. At the electric field strength of 700 V/cm, simulation estimated electric field distribution was not different from the ablation defect seen on follow-up MR imaging (p = 0.43). Simulation predicted cross sectional area of treatment (mean 532.33 ± 142.32 mm^2^) corresponded well with the treatment zone seen on MR imaging (mean 540.16 ± 237.13 mm^2^). Simulation results did not suggest injury to the rectum or neurovascular bundle, matching clinical follow-up at 3 months. Computer simulation estimated zone of irreversible electroporation in the prostate at 700 V/cm was comparable to measurements made on follow-up MR imaging. Numerical simulation may aid treatment planning for irreversible electroporation of the prostate in patients.

## Background

The detection of prostate cancer has shifted to an earlier point in disease development, resulting in the increased incidence of early-stage small-volume cancer. Consequentially, there has been an emergence of minimally invasive surgical interventions designed to provide appropriate local oncologic control with negligible treatment related effects on quality of life (van den Bos et al. 2014; Valerio et al. 2014). Such focal tissue ablation techniques intend to preserve erectile, urinary and rectal function by minimizing damage to anatomical features such as neurovascular tissues, the urinary sphincter, bladder and rectum that surround the prostate. Thermal ablation techniques such as cryoablation (Cytron et al. 2009; Bahn et al. 2012; Onik et al. 2008), high intensity focused ultrasound (HIFU) (El Fegoun et al. 2011; Baco et al. 2014; Crouzet et al. 2014) and focal laser ablation (Lindner et al. 2010) have been evaluated for treatment of patients with localized prostate cancer with good short term outcomes (Ahmed et al. 2011, 2012). However, literature suggests that even focal ablation requires careful planning and application to avoid injury to vitally healthy tissue in the treatment vicinity (Barret et al. 2013).

Irreversible electroporation (IRE) is a new type of focal ablation that uses short high voltage electric pulses to create persistent micropores in the plasma membranes of cells, leading to cell death. IRE has been evaluated for ablation of the prostate in the preclinical (Onik et al. 2007; Neal et al. 2013) and clinical setting (Neal et al. 2014; Valerio et al. 2014). Even though the energy used during IRE may produce heating in the immediate vicinity of the electrodes (Faroja et al. 2013), it has been observed to be safe for the focal ablation of tumors adjacent to heat sensitive structures such as the bile duct (Silk et al. 2014) and the pancreas (Bower et al. 2011) in humans. IRE is performed by placing needle electrodes in tissue and applying a voltage between the electrodes to generate an in vivo ablative electric field. The in vivo electric field distribution is determined by the geometry of ablation probe placement, the voltage applied between the electrodes and the electrical conductivity of the tissue receiving treatment. The therapeutic efficacy and treatment outcomes following IRE ablation is therefore contingent on the size, shape and consistency of the in vivo distribution of this ablative electric field. The ablative electric field used to induce IRE in tissue has been reported to be susceptible to heterogeneities in electrical conductivity in the treatment region (Ben-David et al. 2013). There is concern that such intrinsic redistribution of the ablative electric field may cause unintended safety effects and also alter the intended volume and shape of the final treatment region. Patient specific computer simulations can model the ablative electric field distribution in the prostate using data obtained from pre and intra-operative imaging, and the treatment parameters. Therefore, the purpose of this study was to determine whether retrospectively constructed patient specific numerical simulations can map the treatment effect seen on follow-up MR imaging after irreversible electroporation of patient prostate.

## Methods

This retrospective single institution study was approved by the institutional review board and performed in compliance with the Helsinki Declaration.

### Patients and treatment

Data of six consecutive patients treated with IRE in 2013 (mean age 66.5 years; range 61–70 years) with biopsy proven prostate carcinoma was used in this retrospective study. Focal ablation was performed within 3–6 months of biopsy and 4–6 weeks following MRI. IRE was performed after insertion of needles spaced 10–15 mm apart through a transperineal approach under TRUS guidance. Voltages used for treatment delivery was chosen to achieve an effective electric field strength of 1600–1800 V/cm between any pair of ablation probes used for treatment delivery, seventy 90 μs pulses were used to perform the treatment. Patient characteristics and ablation data are summarized in Table 1. All treatments were performed under general anesthesia with intravenous muscle blockade to reduce electric pulse induced muscle contraction.

**Table 1:** Patient characteristics and ablations data

Clinical history					IRE treatment information				
Patient	Age (years)	History	Stage	Gleason	Number of probes	Mean probe spacing (mm)	Mean voltage (V)	Pulse length (μs)	Number of ablations
1	68	Active surv (2009)	T1c	6 (3 + 3)	4	15 (11–19)	2330 (1650–2850)	90	4
2	70	New Diag.	T1c Apex	6 (3 + 3)	5	13.7 (11–19)	2103 (1650–2850)	90	7
3	64	New Diag.	T1c Apex right	6 (3 + 3)	3	13.3 (13–14)	2000 (1950–2100)	90	3
4	61	New Diag.	T1cApex right	6 (3 + 3)	4	12.2 (10–14)	2075 (1700–2340)	90	4
5	70	Radioth 2008	T2a PZ right	9 (4 + 5)	5	14 (11–16)	2301 (1760–2720)	90	7
6	66	New Diag.	T1c Apex	6 (3 + 3)	3	14 (13–15)	2520 (2340–2700)	90	3

Preoperative MR imaging and intra-operative axial TRUS acquisitions were recorded, with measurement of the prostate in the axial cross-section. Therapy target was guided by location of positive biopsy findings, and by imaging in two patients who had tumors visible on MR imaging. Treatment was planned to achieve 5 mm ablation margin encompassing the region suspicious for malignancy. Follow-up MRI was performed within 21 days (±9 days) after IRE treatment. Post-treatment, all patients underwent follow up imaging in a tertiary referral center on a 1.5 T system (GE, Milwaukee, IL, USA) with an endorectal coil and contrast injection. The MR protocol used for image acquisition is reported in Table 2. Patient reported outcomes regarding urinary and sexual function were obtained using a questionnaire at their clinic visit, along with PSA measurements within 4–6 months after treatment.

**Table 2:** MR imaging protocol used during follow-up imaging

MRI protocol	MRI Sequences			
	T1 weighted	T2 weighted	DWI	Dynamic
	SE	SE	EPI	GE
Plane	Axial	Axial	Axial	Axial
Time to repeat (ms)	646	6450	4500	3.77
Time to echo (ms)	12	116	91	1.46
Angulation (°)	134	170		10
Thickness (mm)	3.5	3.5	3.5	3.5
FOV (mm)	160	150	180	190
Matrix (mm × mm)	246 × 256	246 × 256	102 × 128	128 × 160
Scan time (s)	150	390	340	260
Time resolution (s)	–	–	–	10

*MRI* magnetic resonance imaging, *DWI* diffusion weighted imaging, *FOV* field of view, *SE* spin echo, *EPI* echo planar imaging, *GE* gradient echo

### Image processing

**Fig. 1:**
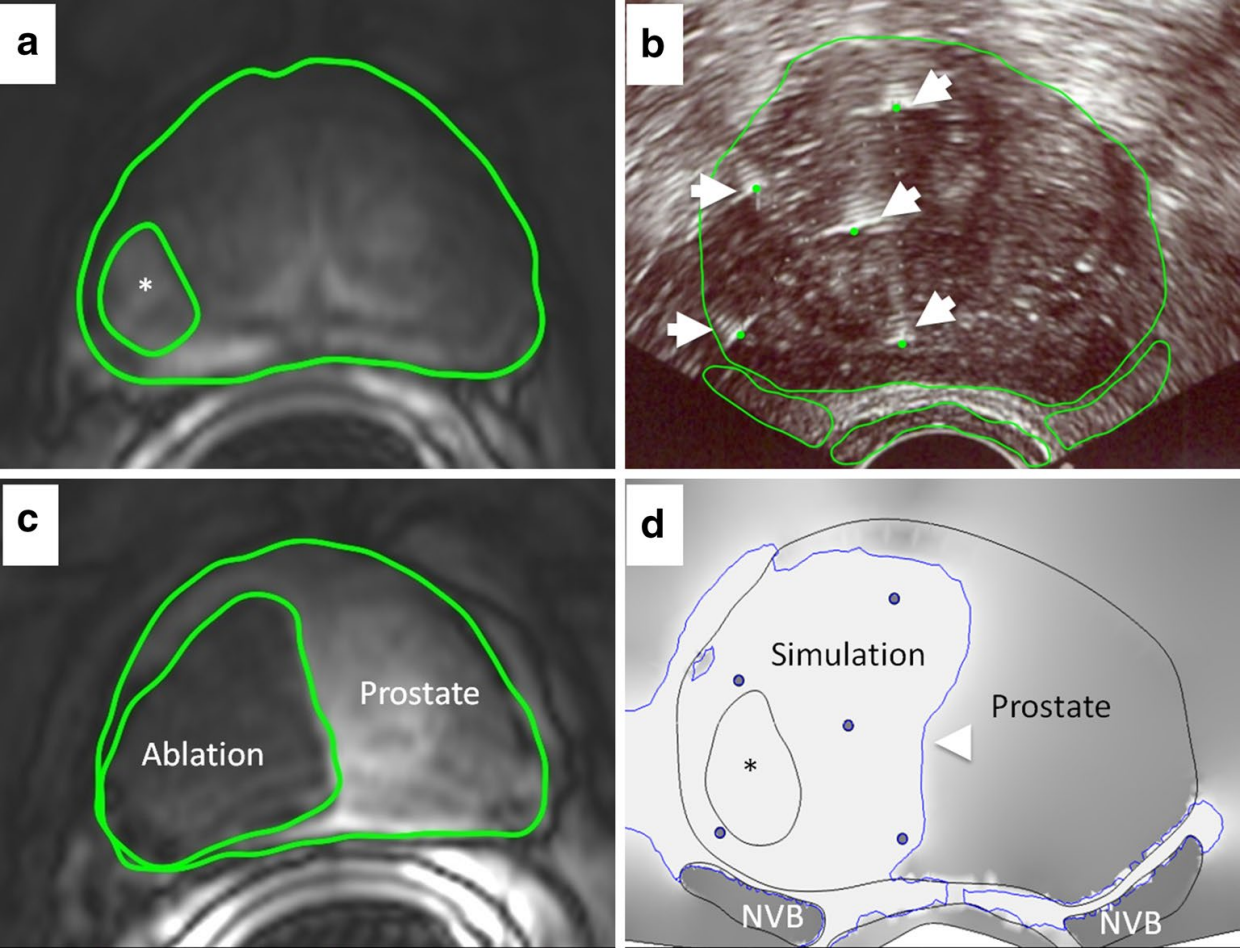
Overview of workflow used to perform patient specific simulation for IRE treatment performed on a 70-year-old man with Gleason 9 recurrence after radiation therapy (patient 6). **a** T1 weighted post contrast MRI showed a tumor (*center marked with an* *asterisk, boundary with solid line*) in the right peripheral zone. **b** Intra-operative axial US guided needle placement to the tumor (5 IRE needles, *white arrows*). The intra-operative US image annotated to demarcate the ablation probes (*dashed arrows*), the outline of the prostate, the rectum, and the neurovascular bundles (NVB) (*solid lines*). Clinical treatment planning data were compiled and the MR images from the corresponding axial plane were used to identify critical structures for segmentation. **c** Follow-up axial enhanced T1w with fat saturation MR imaging performed 15 days after ablation was used to demarcate the ablation defect (*solid line*) and showed size and shape of the ablative zone (area: 701 mm^2^). **d** Simulation predicted ablation zone (*white with blue* boundary) at the electric field strength contour (700 V/cm; area 624 mm^2^, *arrowhead*). Image plotted using gradient shading with regions of highest field strength (700 V/cm and stronger) appearing *light* and lower field strengths appearing *dark*. Simulation predicted that ablation encompassed completely the tumor

The intra-operative ultrasound image in the axial plane corresponding to the midpoint of the treatment zone (mid-point of ablation volume) was manually registered with the pre-operative MR images. The registered image set was then annotated to demarcate the ablation probes, the outline of the prostate, rectum, neurovascular bundles and the tumor (where observable) using OsiriX DICOM Viewer (Fig. 1a, b). This annotated image set was used to generate the 3D models for the simulation.

Subsequently, the follow-up MR images were also registered with the intra-operative US images in a similar process. The ablation defect was segmented from the follow-up imaging. The area of the prostate was measured from the intra-operative US images, and the follow-up MR images using GNU image processing software (GIMP). The area of the ablation defect was measured from the follow-up MR image and simulation results. In addition to visual assessment performed by two radiologists, the influence of post-treatment edema and resulting error in registration were evaluated by comparing the ratio of the prostate’s area between intra-operative US images and the follow-up MR images. The ablation area measurements from the follow-up MR image and the simulation results were compared in a similar fashion.

### Simulation

A numerical model of the Laplace equation was solved to estimate the electric field distribution within the tissue due to application of voltage between pairs of needle electrodes used for treatment delivery. Techniques previously described by Neal et al. (2012, 2010) and Daniels and Rubinsky (2012) were used to set up and solve the numerical models. The modeling and simulation techniques were developed and validated partly using data generated from IRE treatments performed in the kidney and pancreas of a large animal model (Wimmer et al. 2013, 2015).

Finite element computer simulations were used to estimate in vivo distribution and magnitude of the ablative electric field within the prostate and surrounding anatomic structures. The annotated intra-operative US images were imported into Inventor (Autodesk, San Rafael CA), and the data was manually segmented. The segmented regions were then used to generate 2.5 mm thick 3D geometry for each region identified in the US image. This specific slice thickness was chosen to roughly correspond to slice thickness (3.5 mm) and spacing used during acquisition of the post-operative MR images (Fig. 1c). The 3D models created in Inventor were imported into Comsol (Comsol Inc., MA) and discretized into a finite element mesh. Material properties of biological tissue used for performing the simulation are described in Table 3. The simulation was performed for a single pulse applied between pairs of electrodes used for treatment delivery in patients, and the electric field resulting from each such application was used to arrive at the cumulative electric field distribution at the end of treatment. Isoelectric contours were drawn at 100 V/cm intervals (500–1500 V/cm) for comparison with follow up MR imaging. The isoelectric contours were used to identify the electric field threshold (Fig. 1d) that best matched with the ablation defect seen on follow-up MR imaging (Fig. 1c).

**Table 3:** Tissue electrical properties used in numerical simulation

Tissue type	Electrical conductivity S/m	References
Healthy prostate	0.41	Neal et al. (2014), Daniels and Rubinsky (2012), Halter et al. (2009)
Prostate tumor	0.3	Neal et al. (2014), Daniels and Rubinsky (2012), Halter et al. (2009)
Axon	1.44	Daniels and Rubinsky (2012)
Fat	0.012	Daniels and Rubinsky (2012)
Blood	0.7	Daniels and Rubinsky (2012)
Muscle	0.2	Daniels and Rubinsky (2012)
Colon	0.01	Daniels and Rubinsky (2012)

### Statistical analysis

Patient data was compiled from a review of all medical, imaging and pathological reports. Results of simulation studies were compared to outcomes determined by clinical data (PSA, quality of life survey) as well as follow-up MR imaging. The size of the ablation as estimated by numerical simulation and the actual ablation defect seen on MR imaging was compared and statistically analyzed for significance. Our study attempts to demonstrate that simulation model estimated ablation defect will not be different (or non-inferior) to the ablation defect measured on MR imaging. This requires a total of four samples per group to determine non-inferiority at 95 % confidence interval. In our case, the control (MRI) and treatment (simulation) data is drawn from the same patient, and our study was conducted using six patients (25 % more than what is required to achieve statistical power). Statistical analysis was conducted with Fisher exact test, χ^2^ for independence test (Excel; Microsoft, Redmond, WA) and p < 0.05 was considered significant. The ablation zones from the simulation and MR imaging were also evaluated using Pearson product moment correlation.

## Results

### Imaging outcomes

In the immediate post-treatment setting, non-contrast US images indicated presence of multiple diffuse regions with hyperechoic appearance within the ablation zone. There was also the occasional appearance of a hyperechoic region at the margin of the planned treatment zone. However, this was not clearly present in the entire treatment region and was not observed in all patients.

**Fig. 2:**
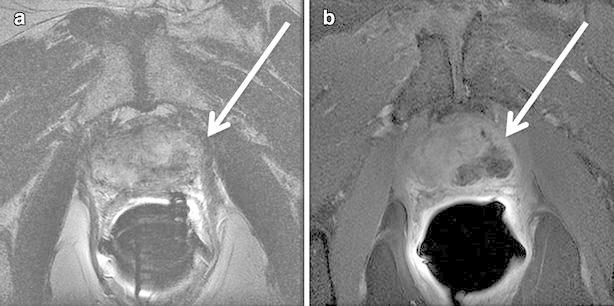
Typical findings on follow-up MRI 3 weeks after IRE of the prostate. From one patient, **a** the treatment zone appears as heterogeneous hyperintense region (*arrow*) with regions of low signal intensity. **b** The lesion (*arrow*) is easily visualized on contrast enhanced T1w imaging

It was possible to discriminate between treated and untreated regions of the gland on follow-up MR imaging. The expected treatment zone appeared as a heterogeneous hypointense zone on T1 imaging, but it was not possible to clearly separate the treated region from surrounding normal gland. On T2 imaging, the ablation zone appeared as a hyperintense region interspersed with low intensity locations that were consistent with ablation probe tract (Fig. 2a). On contrast-enhanced dynamic T1 imaging performed with fat suppression, the treatment region appeared non-enhancing with limited to no enhancement of tissue in the periphery (Fig. 2b). The T2 and contrast-enhanced dynamic T1 images were used to identify and segment the expected ablation zone for comparison with simulation results.

It was possible to obtain good registration of the intra-operative US and the follow-up MR imaging. The following results were obtained from computing the ratio of prostate’s area as measured with the two modalities at the registered slice (mean 0.99, range 0.86–1.07). The measured area of the prostate from the two modalities were not significantly different (p = 0.49, see Table 4).

**Table 4:** Area of prostate (mm^2^) observed with MRI and US at the same level

Patient	Axial cross-sectional area of prostate (mm^2^)		Axial cross-sectional area of ablation zone (mm^2^)	
	MRI	US	MRI	Simulation
1	2055	1916	590	585
2	1966	1877	849	735
3	1420	1633	210	340
4	888	895	322	449
5	1309	1377	701	624
6	1741	1682	569	461
Mean (SD)	1563.16 (±441.98)	1563.33 (±380.28)	540.16 (±237.13)	532.33 (±142.32)
*p*	Not significant: (0.49)		Not significant: (0.43)	

Size of the prostate ablation observed with MRI and based on simulation (with a threshold level of sensitivity of 700 V/m^2^)

Axial cross sectional area of the prostate of the post-treatment MRI and US were compared to validate the accuracy of registration. The US and simulation image are at 1:1 scaling

### Simulation outcomes

**Fig. 3:**
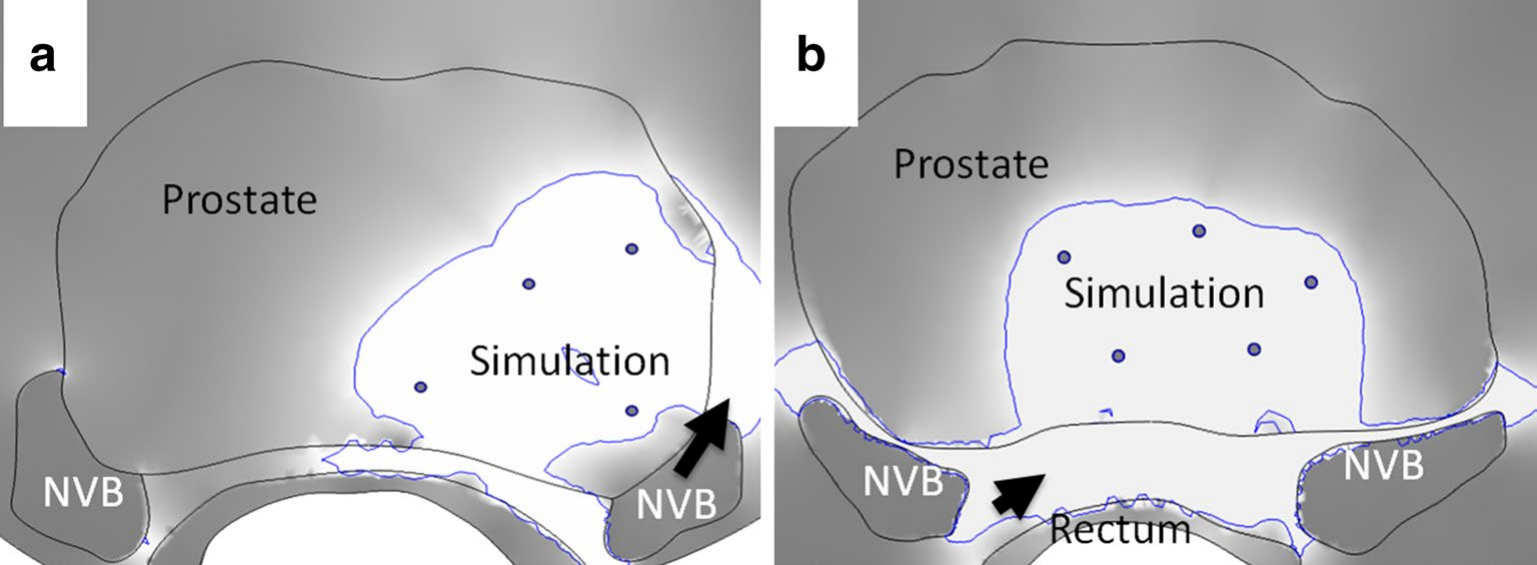
Simulation findings suggest that the ablative electric field was not restricted to the prostate, and was seen penetrating peri-prostatic fat and muscle tissue. Neurovascular bundle and rectal tissues may be drawing the ablative electric field towards them and thereby affecting the size and shape of the ablation within the prostate. **a** The neurovascular bundle influenced the shape of the electric field in ablations performed in the periphery of the prostate (*arrow*) (patient 1). **b** The rectum was seen to be influencing the electric field of ablations performed centrally in the prostate (*arrow*)

**Fig. 4:**
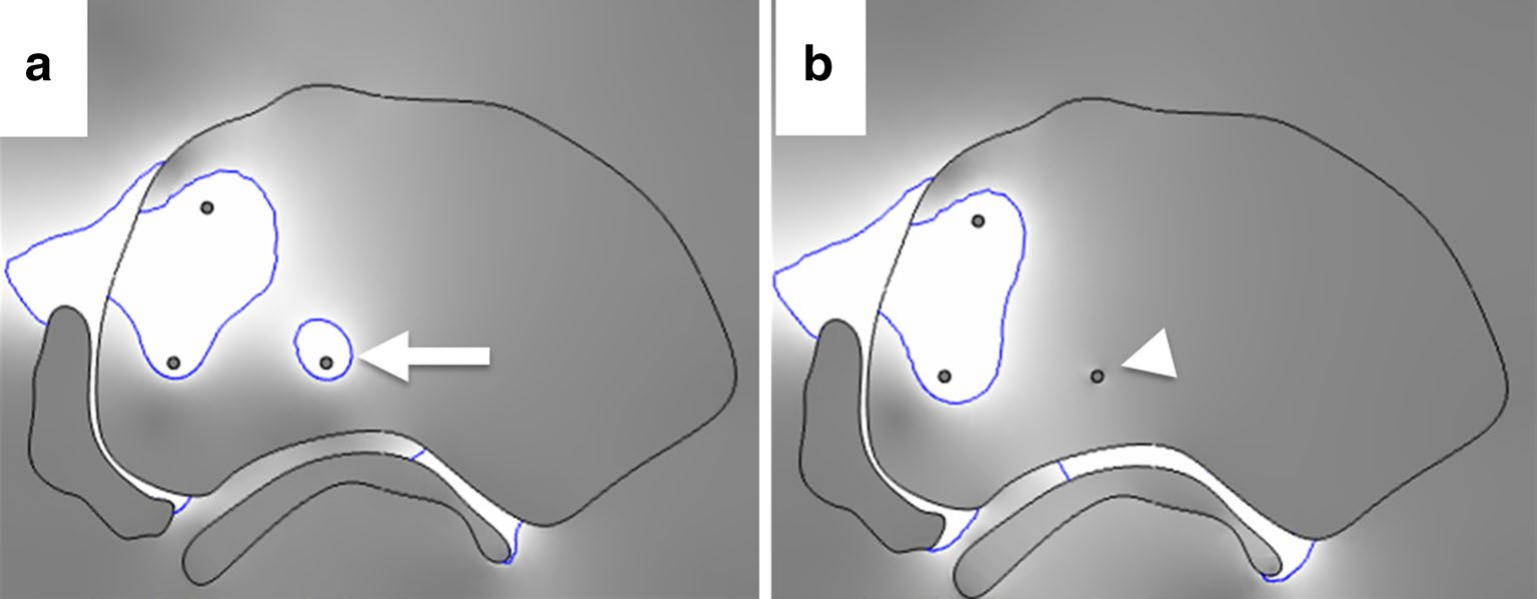
The effect of exposed but unused ablation probes on ablation outcomes. **a** Simulation representing actual clinical scenario where unused ablation probes are left exposed in the prostate while ablation is delivered through the other pair of ablations probes (*white arrow* indicates current drawn around un-insulated probe not used for ablation) (patient 4). **b** Simulation representing scenario if the unused probe had been insulated prior to delivering ablation between the other two probes (*white arrow*)

Individualized models were created and simulations performed for every patient included in this study (for example; Fig. 1). Simulation findings suggest that the ablative electric field was not restricted to the prostate, and was seen penetrating peri-prostatic fat and muscle in all patients (Fig. 3). In all simulations, the maximum electric field strength in sensitive structures adjacent to the prostate, such as the neurovascular bundle (<300 V/cm) or the rectum (<600 V/cm), were at levels at which minimal or no IRE induced damage was expected at these structures. An electric field gradient was developed within the prostate from application of voltage between electrodes, and the electric field was strongest in the immediate vicinity of the ablation probes. The electric field was estimated to completely cover the tumor in the two cases where tumors were identifiable on pre-operative MR images. Simulations estimated an irregular and non-convex ablation zone in all patients. None of the ablations performed between any pair of needle electrodes was observed to have a regular convex ellipsoidal shape in the axial cross section. Metallic objects in the vicinity of the treatment zone were observed to influence the electric field distribution. This effect largely appeared with exposed ablation probe tips present in the treatment region, but not actively used for energy delivery (Fig. 4; energy delivery was performed between one pair of ablation probes at a time).

### Comparison of simulation and follow-up imaging

The contour plot of electric field strength from the simulation was registered and compared with follow-up MR images to identify the electric field strength threshold that matched with the size of the post-ablation defect on imaging. It was found that the electric field strength contour at 700 V/cm correlated closely with the follow-up MR images (Simulation predicted ablation area: 532.33 mm^2^ in mean for all patients vs Ablation area measured on imaging: 540.16 mm^2^ in mean, p = 0.43) for all patient cases reviewed. This threshold was used to demarcate the expected ablation zone within the prostate and measurements (area and cross section) were performed using GIMP and presented in Table 4. Radiologist interpretation from two separate observers (F.C. and H.T.) suggested that the shape and size of the ablation predicted by the simulation compared well with all post-operative MR imaging in all cases. The two measurements had a correlation coefficient of 0.945 when evaluated using Pearson product moment technique. The ratio of measurements taken from the two modalities also indicated good correspondence between the techniques (mean 0.97; range 0.61–1.2).

### Patients reported outcomes

Evaluation of the self reported forms suggested that none of the patients undergoing IRE therapy developed impotence or urinary incontinence following treatment. A significant decrease of PSA (p = 0.01) was observed in all patients during clinical follow-up. Clinical outcomes are summarized in Table 5.

**Table 5:** Clinical outcomes following treatment

Patient	Follow-up MRI (days)	Post-op biopsy (outcome)	Most recent post-op PSA (months)	PSA (pre) (ng/ml)	PSA (post) (ng/ml)	Potent	Continent
1	35	+ve	7	3.96	3	Yes	Yes
2	23	+ve	11	1.9	1.24	Yes	Yes
3	29	+ve	6	5.59	4.44	Yes	Yes
4	22	−ve	7	1.71	1.12	Yes	Yes
5	15	−ve	8	5.63	1.1	Yes	Yes
6	10	−ve	9	5.42	3.15	Yes	Yes
Mean (SD)	22.3 (±9.07)		8 (±1.78)	4.03 (±1.83)	2.17 (±1.59)		

## Discussion

Irreversible electroporation for the focal ablation of prostate is a new treatment technique and has seen use at just select centers in the world (Valerio et al. 2014). The ability to perform non-thermal treatment with IRE makes it a valuable clinical alternative to existing thermal ablation techniques such as HIFU or cryotherapy. However, there exist considerable knowledge gaps on the typical imaging findings, and treatment outcomes following IRE of prostate, and this has restricted wider use of this technique. Currently, it is not possible to directly measure the electric field strength generated in the targeted tissue during IRE. Therefore unlike HIFU, where MR imaging can be employed to monitor temperatures and subsequently estimate the effective treatment zone, there is no simple way to predict the expected ablation zone following IRE. Therefore treatment planning may be crucial for guiding the safe delivery of IRE in patients. Our results demonstrate the feasibility of using numerical simulations constructed with pre-operative MR images and intra-operative US to estimate the treatment zone, which was comparable to what was observed on follow-up MR imaging. In the future, such simulations could potentially be used to plan and guide IRE treatment in patients in a prospective fashion.

As a focal ablation technique, post-treatment imaging findings are crucial to understand tissue ablation with IRE, and also to prognosticate treatment outcomes. MRI findings have been previously reported for the acute effects and short-term injury evolution following IRE in different animal models, including work by Zhang et al. (2014) in rats with hepatoma, and Wendler et al. (2013) in normal swine kidney. There is limited published information on the typical imaging findings following IRE in the prostate and while not the primary focus of this work, we report our imaging findings. Consistent to prior reports from animal models studies, MR imaging following IRE presents as a heterogenous region with both hyper and hypointense regions on T1 and T2w imaging. The ablation zone was best visualized using contrast enhanced T1w imaging, appearing as a region of non-enhancement when compared to untreated gland. Also, imaging findings observed by us on US following IRE were similar to previously reported findings after IRE in solid organs (Schmidt et al. 2012). However, further study is warranted before these findings can be translated into clinical meaningful results.

The ablation boundary estimated at the threshold field strength of 700 V/cm in simulations correlated well with the post-operative MR images from our patients. Prior reports from in vivo studies performed on healthy dog prostate suggest irreversible damage to cells in regions that experienced electric field strength of 600 V/cm or higher (Onik et al. 2007). A study performed by Qin et al. (2013) using a LNCaP tumor model suggests that the critical field strength required for achieving irreversible contingent can range from 600 to 1300 V/cm, and will vary based on the number of pulses applied and the pulse duration. Our results are in agreement with these prior studies. However, a study performed by Neal et al. (2014) with simulation-pathology correlation of human prostates resected 3–4 weeks following IRE ablation reported a higher threshold for IRE induced cell death (1072 V/cm). While this study used volumetric modeling of the ablation zone and a different simulation technique, the study results are restricted by the small dataset (two patients) used for their simulations. Since we did not have pathology following ablation from the patients enrolled in this study, 700 V/cm as the critical threshold is drawn purely in correlation to MR imaging information. A larger study may be required before the differences between imaging and pathology measurements can be clarified, and until such time our findings should only be taken in context of post-IRE MR imaging of the prostate.

Confirming findings from the large animal study reported by Ben-David et al. (2013), the in vivo distribution of the ablative electric field that induces IRE in the tissue was found susceptible to regional heterogeneities in electrical conductivity. During IRE treatment all ablation probes to be used for the treatment are inserted together in a pre-determined configuration along the long axis of urethra, and subsequently treatment is delivered between select pairs of ablations probes. Simulation electric field maps suggest that the electric field may get drawn towards the exposed tips of the unused probes (Fig. 4).

Our retrospective patient-specific simulations suggested lack of injury to the neurovascular bundle and rectum in our patient cohort, and these findings were reflected in the patient reported outcomes. Our simulation results suggested that IRE mediated tissue destruction was largely restricted to the prostate. Despite being in proximity to the ablation zone, simulation indicated the sparing of the rectum, neurovascular bundle and the urethra. In agreement with simulation findings, no impotency or incontinence has been reported during a mean follow up time of 8 months following treatment.

Our study has a few limitations, primarily the exploratory nature of the work and the small number of patients enrolled in this study. These factors may limit the generalization of our findings. The simulation was performed using data segmented from intra-operative US images, which has less information than the pre-operative MR images. Complex and heterogeneous structures such as the neurovascular bundle were represented in the model as simpler lumped structures with homogenous electrical properties which reduce precision of the simulation model. Our simulation was not truly volumetric and was restricted to a single thick slice of tissue and therefore may not estimate the behavior of the entire ablation volume. Our results are pertinent for comparison of simulation findings with post-treatment imaging, and the absence of pathology data limits drawing definitive conclusions on the status of the treated tissue. Post-ablation tissue is constantly evolving, undergoing edema and tissue expansion in the early phase followed by fibrosis and tissue shrinkage at later periods. These dynamic changes may explain some of the inter-patient differences in simulation versus MRI measurements we observed. Finally, gadolinium-enhanced MRI by itself is not a validated technique for measuring the effectiveness of IRE in the prostate; therefore, we are unable to arrive at any conclusion on the value of simulations for estimating true treatment outcomes.

In summary, our results suggest that simulation of IRE ablation matches treatment zones seen on MRI, and therefore may help in treatment planning of ultrasound-guided IRE in the prostate. While we provide evidence that simulations can be used to estimate the size and shape of the expected IRE ablation in patient prostate with good correlation to MR imaging, further comparison with pathology are required before using simulations to predict treatment efficacy.

## Conclusions

It is feasible to use mathematical modeling and simulation to estimate the effects of irreversible electroporation in patient prostate. Simulations estimated size and shape of treatment zone correlates with ablation defect seen on MR imaging following IRE of the prostate. Isoelectric contour at 700 V/cm on simulation can be used to determine expected treatment effect as seen on post-treatment MR imaging.
